# Modified Xiaoyaosan (MXYS) Exerts Anti-depressive Effects by Rectifying the Brain Blood Oxygen Level-Dependent fMRI Signals and Improving Hippocampal Neurogenesis in Mice

**DOI:** 10.3389/fphar.2018.01098

**Published:** 2018-09-28

**Authors:** Lei Gao, Peng Huang, Zhaoyang Dong, Tingting Gao, Shaohui Huang, Chuying Zhou, Yuling Lai, Guanghui Deng, Bin Liu, Ge Wen, Zhiping Lv

**Affiliations:** ^1^School of Traditional Chinese Medicine, Southern Medical University, Guangzhou, China; ^2^School of Nursing, Guangzhou University of Chinese Medicine, Guangzhou, China; ^3^Zhujiang Hospital, Southern Medical University, Guangzhou, China; ^4^Nanfang Hospital, Southern Medical University, Guangzhou, China

**Keywords:** depression, fMRI, traditional Chinese medicine, hippocampus, blood oxygen level-dependent

## Abstract

As the traditional Chinese herbal formula, Xiaoyaosan and its modified formula have been described in many previous studies with definite anti-depressive effects, but its underlying mechanism remains mystery. Previous work in our lab has demonstrated that depression induced by chronic stress could generate brain blood oxygen level-dependent (BOLD) functional magnetic resonance imaging (fMRI) signals disorder, accompanied by the impairment of hippocampal neuronal plasticity, decrease of brain-derived neurotrophic factor, and reduction of the number and complexity of adult neurons in the dentate gyrus. We hypothesized that herbal formula based on Xiaoyaosan could exert anti-depressive effects through restoring these neurobiological dysfunctions and rectifying BOLD-fMRI signals. To test this hypothesis, we examined the effect of modified Xiaoyaosan (MXYS) on depressive-like behaviors, as well as hippocampal neurogenesis and BOLD signals in a mice model of chronic unpredictable mild stress (CUMS)-induced depression. MXYS exerted anti-depressant effects on CUMS-induced depression that were similar to the effects of classical antidepressants drug (fluoxetine hydrochloride), with a significant alleviation of depressive-like behaviors, an improvement of hippocampal neurogenesis, and a reversal of activation of BOLD in the limbic system, particularly in the hippocampus. These results suggested that MXYS attenuated CUMS-induced depressive behaviors by rectifying the BOLD signals in the mice hippocampus. These novel results demonstrated that MXYS had anti-depressive effects accompanied by improving BOLD signals and hippocampal neurogenesis, which suggested that BOLD-fMRI signals in brain regions could be a key component for the evaluation of novel antidepressant drugs.

## Introduction

As a complicated mental and neuropsychological disease, depressive disorder is a serious challenge worldwide (Steel et al., 2014; Greenberg et al., 2015). Exposure to chronic stress is widely regarded as the significant risk factor for the development of depressive disorder. Epidemiological findings indicate that stressful life events frequently precede the onset of depressive disorder in human populations (Sterner and Kalynchuk, 2010). Studies of patients with depressive disorder have appeared dendritic atrophy in hippocampus and prefrontal cortex, as well as reduction of hippocampal volume (Campbell et al., 2004). Depression induced by stress produces neurobiological alterations, along with decreases in brain-derived neurotrophic factor (BDNF) expression and hippocampal neurons proliferation, survival, and maturation (Lussier et al., 2013). In addition, the consequences of depressive disorder include not only focal changes within certain brain regions, but also disturbances of function within them.

Brain regions involved in mood-regulating circuit, which detected by the functional magnetic resonance imaging (fMRI), have demonstrated their roles in the development and progression of depression (Hui et al., 2016; Huang et al., 2018). As shown in our previous fMRI studies, chronic unpredictable mild stress (CUMS) induced depressive behaviors accompanied with up-regulation of blood oxygen level-dependent (BOLD) signals in mouse brain regions, primarily in the limbic system, such as the cortex, hippocampus, and corpus calcium, depending on the amplitude of low-frequency fluctuations (ALFFs) analysis (Huang et al., 2017). Recently, we further found that neural circuitry was connecting the hippocampus, prefrontal cortex, and basolateral amygdala in CUMS-induced depression mice model, which were associated with BOLD–fMRI signals (Huang et al., 2018). Thus, depression may be driven by disorder neural circuits across multiple brain regions, and BOLD–fMRI signal could be an important indicator in depression. Unfortunately, until now, few studies used BOLD analysis to evaluate the antidepressant effects of drug, especially in the study of traditional Chinese medicine (TCM).

In TCM philosophy, depression is caused by disturbances in endogenous physical condition and exogenous pathogenic factors. Holistic enhancement of inner defenses and restoration of normal balance are utilized for depression therapy, which differs from Western medicine. As a traditional Chinese herbal formula, Xiaoyaosan has been classically prescribed for the treatment of mental disorders for thousands of years in China (Ding et al., 2014; Jing et al., 2015). It was originally described in *Taiping Huimin Heji Jufang*, a Chinese materia medica officially compiled in the Song Dynasty of China (960–1127 AD). In our previous reports, Xiaoyaosan and its modified formulas (such as Danzhi Xiaoyaosan) have regulatory effects in CUMS-induced behavioral disorders, along with the mechanism of ameliorating hypothalamic–pituitary–adrenal (HPA) hyperactivation, neurotrophic and neurobiological dysfunction, as well as maintain the balance of the kynurenine (Kyn)/serotonin (5-HT) pathway, and so on (Zhu et al., 2014; Jing et al., 2015; Jiang et al., 2016). For further precise administration of major depressive disorder (MDD) patient, we have established an empirical prescription formula, the modified Xiaoyaosan (MXYS), which consisted of 10 crude herbs: *Radix Bupleuri, Radix Angelicae Sinensis, Radix Paeoniae Alba, Rhizoma Atractylodis Macrocephalae, Rhizoma Acori Tatarinowii, Curcuma Aromatica, Caulis polygoni multiflori, Fructus Schisandrae Chinensis, Semen Ziziphi Spinosae*, and *Os Draconis*. MXYS has been shown to be an effective therapy for the syndrome of liver qi stagnation and spleen deficiency, which is closely related to mental illnesses, especially for the MDD patients in clinic. Although many studies have focused on investigating the mechanism by which herbal formulas reduce depressive-like behaviors, few studies have shown sufficient evidence in exploring the anti-depressant effects of herbs through the regulation of brain function and neural circuitry, particularly the BOLD signal responses in brain regions.

Based on our previous experimental evidences, BOLD signals in the brain are important contributors to the pathogenesis of depressive disorders (Huang et al., 2017, 2018). Therefore, we hypothesized that MXYS could reduce depression by revising the brain BOLD signals and improving adult hippocampal neurogenesis in mice. To test this hypothesis, we utilized the CUMS procedures, which can produce a mice depressive phenotype (Papp et al., 1991). We then assessed the depression-like behavior using the sucrose preference test (SPT), tail suspension test (TST), and forced-swim test (FST). On the other hand, the hippocampal neurogenesis was evaluated by frozen hippocampus tissue, and BODL-fMRI signal was analyzed by ALFF detection. Our results support our hypothesis and suggest that MXYS increases hippocampal BDNF expression and corresponding neurogenesis markers, and normalizes the behavioral phenotype and BOLD signal.

## Materials and Methods

### Animals

All procedures for the animal studies were approved by the National Institutional Animal Care and Ethical Committee of Southern Medical University. The experimental protocols applied in this study were performed in accordance with the approved guidelines. Eight-week-old male C57BL/6J mice (Animal Experimental Center, Southern Medical University, China) were randomly divided into different groups (*N* = 10): (1) control group, (2) CUMS group, (3) MXYS treatment group, and (4) the classical antidepressants drug (fluoxetine hydrochloride, FH) treatment group. All animals were housed in an animal facility where temperature and humidity are controlled (21 ± 2°C and 55 ± 5%) with a light–dark cycles for 12:12 h alternately. The body weight and consumption of food and water of each mouse were recorded weekly.

### CUMS Procedure

**FIGURE 1:**
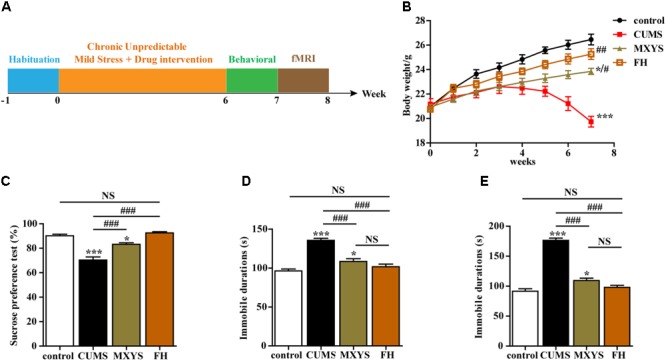
Effects of MXYS on depressive-like behaviors. **(A)** Schematic representation of the experimental design used for this study. All animals received the same amount of handling, behavioral testing, and fMRI detection. **(B)** Body weight gain. The body weights of mice were recorded weekly. **(C)** Effects of the CUMS treatment on sucrose preference. **(D)** Immobility time in the TST. **(E)** Immobility time in the FST. The data are presented as means ± SEM. ^∗^*P* < 0.05, ^∗∗∗^*P* < 0.001 versus control group; ^#^*P* < 0.05, ^##^*P* < 0.01, ^###^*P* < 0.001 versus CUMS group; NS, *P* > 0.05.

Briefly, the CUMS procedure involved nine different stress events, which were randomly arranged throughout 42 consecutive days. The stressors were (1) fasting and water deprivation for 24 h, (2) exposure to an empty bottle for 1 h, (3) 45° tilting cage lasts for 17 h, (4) overnight illumination, (5) exposure to a wet cage for 24 h, (6) swimming in cold water (4°C) for 5 min, (7) disrupting the cage for 24 h, (8) foreign body stimulation lasts for 24 h, and (9) 4 h of restricted movement. Schematic representation of the experimental design is depicted in **Figure 1A**.

### Preparation of MXYS

Modified Xiaoyaosan consists of 10 herbs: *Radix Bupleuri, Radix Angelicae Sinensis, Radix Paeoniae Alba, Rhizoma Atractylodis Macrocephalae, Rhizoma Acori Tatarinowii, Curcuma aromatica, Caulis polygoni multiflori, Fructus Schisandrae Chinensis, Semen Ziziphi Spinosae*, and *Os Draconis*. The putative major ingredients and targets of 10 herbs in MXYS are listed in **Supplementary Table 1**. Following the Regulation on Processing of Traditional Chinese Medical Herbal Pieces, all of the raw herbs were purchased from Nanfang Hospital and processed into a no decocted extract in EFONG Pharmaceutical Company (Guangdong, China). The production process of the no decocted MXYS was described in **Supplementary Figure 1**. The quality of MXYS was controlled by the regular items under the 2010 edition of the Pharmacopeia granules. The drug was packaged in a medicinal composite film, which is in Line with the pharmaceutical packaging specifications. Meanwhile, for the quality control (QC), we have identified critical compounds from MXYS samples employing an HPLC-LTQ-Orbitrap-MS eluted system, which matched the corresponding peaks in MXYS. Details were conducted as described in the **Supplementary Materials, Supplementary Figures 2–4**, and **Supplementary Tables 2,3**.

### Drug Administration

In our experiments, the drug dose was converted according to the human and animal equivalent dose formula and administered by gavage once a day for 6 weeks. We dissolved MXYS (10 g) in 250 ml distilled water to a final concentration of 0.04 g/ml. The animal was treated with MXYS under the dosage of 0.4 g/kg B.W., and the FH group was given FH (0.2 mg/ml, 0.1 ml/mice), while as a comparison the control group and CUMS group were given the same dose of normal saline.

### Behavioral Tests

The behavioral tests were performed and scored by three trained and experienced observers who were blinded to the treatment conditions. All animals were subjected to the behavioral tests at the end of the 6th week.

#### Sucrose Preference Test

The SPT performed at the end of CUMS procedure follows the previous study (Can et al., 2012). In the beginning of the test, animals were exposed to 1% sucrose solution (w/v) for 24 h to habituate to drink sucrose solution. Afterward, two bottles contained with 1% sucrose solution or pure water, respectively, were holding in the cage for mice consumption. After 24 h, the consumption volume of the each solution was recorded and analyzed with the formula: percentage sucrose preference = (sucrose solution consumption/total liquid intake) × 100%.

#### Tail Suspension Test

Briefly, TST was defined as the mice were hung in the hanging box by binding their tail, and the whole process was recorded by video camera. After that, the total immobile time of mice against the tail suspension was recorded and scored. Small movements were not regarded as mobility, which is confined to only the mobility bouts of front limbs with momentum-induced oscillations and pendulums.

#### Forced Swimming Test

Forced swimming test (FST) was a widely used test to probe depression-like behaviors in mice. In short, a plastic cylinder (height: 50 cm, diameter: 38 cm) containing 38 cm of water (25 ± 1°C) was used to hold the animal, in the same time, the whole process (6 min) was recorded by video camera. The immobility time of the animals in water will be scored by three observers, who were blinded to the treatment conditions. Immobility was defined as animals floated without movement or just with small movements, which was only to keep the head above water. The immobility time under the swimming testing was adopted as an indicator of behavioral despair for animals (Russo et al., 2013).

### fMRI Data Acquisition

Functional magnetic resonance imaging scan was performed after behavioral tests as previous studies (Huang et al., 2018). Animals were anesthetized by isoflurane with two stages: the induction stage (a ratio of 1% oxygen + 3% isoflurane) and the continuous anesthesia stage (0.5% oxygen + 2.5% isoflurane). Then, mice were fixed to minimize head motion by a custom-made MRI-compatible cradle with ear and bite bars. A pressure-sensor (SAII Instruments, Model 1030 Monitoring & Gating System, United States) was used to monitor the body temperature and respiration rates. We also adjusted the depth of anesthesia of mice to maintain a consistent level according to the respiration rate.

Functional magnetic resonance imaging was performed on a 7T Animal MRI Scanner (70/16 PharmaScan, Bruker Biospin GmbH, Germany), which was with the configuration of a 38-mm birdcage rodent brain quadrature resonator for radiofrequency transmission and reception. Echo-planar imaging (EPI) with a low spatial and high temporal resolution were used for acquiring the phase images. Parameters followed the previous studies (Huang et al., 2018): protocol = ax-T1w, resolution = 0.14 mm × 0.14 mm × 1.0 mm, matrix size = 192 × 128, slice thickness = 1.40 mm, slice gap = 0.10 mm, repetition time = 603.94 ms, echo time = 9.01 ms, averages = 32, scan time = 5 m 10 s, repetitions = 1, and volume = 1. A statistical parametric mapping (SPM) toolbox (plug-ins) named SpmratIHEP toolki was used to preprocess the phase images (Nie et al., 2010, 2013, 2014).

### Western Blot Analysis

The expression of the BDNF protein in the hippocampus was detected by western blotting. Firstly, the hippocampal tissues were homogenized in radioimmunoprecipitation assay (RIPA) buffer and the supernatants were separated and collected. Concentrations of the protein were determined by the bicinchoninic acid (BCA) kit (Thermo Fisher Scientific, United States). Proteins were separated through 11% SDS–PAGE gels and then transferred to a nitrocellulose membrane. After blocking with 5% fetal bovine serum albumin for 2 h, the membranes were incubated with primary antibodies overnight at 4°C. After washing by TBST buffer, the membranes were incubated with the appropriate HRP-conjugated secondary antibodies. After that, the bands corresponding to the proteins of interest were visualized by enhanced chemiluminescence reagent (Thermo Fisher Scientific, Waltham, MA, United States) and subsequently scanned. Finally, analysis software Quantity One (Bio-Rad Laboratories, Hercules, CA, United States) was used for the band densities detection and calculation. The antibodies used in the present study are listed in **Supplementary Table 4**.

### qPCR Analysis

The levels of the BDNF mRNA in the hippocampus were detected using qRT-PCR. High capacity RNA-to-cDNA kit (TaKaRa, Shiga, Japan) was used to synthesize the cDNA templates according to the manufacturer’s protocol. The sequences of primers for RT-PCR followed our previous study (Huang et al., 2018) and were described as follows: BDNF forward: 5′-TCATACTTCGGTTGCATGAAGG-3′, reverse: 5′-AGACCTCTCGAACCTGCCC-3′; GAPDH forward: 5′-AAGGGCTCATGACCACAGTC-3′, reverse: 5′-GGATGCAGGGATGATGTTCT-3′. In each sample the relative mRNA expression was normalized to GAPDH and the difference compared to the control sample was quantified. The products were analyzed by densitometry with the Multiplex Quantitative PCR System (MX3005P^TM^, Stratagene, United States).

### Immunofluorescence (IF) Staining

Firstly, hippocampus tissues were first fixed with 4% PFA at 4°C for 24 h and sliced into 40-μm thick coronal sections. Secondly, 0.3% Triton X-100 was used for the permeabilization of free-floating sections. After that, the sections were blocked in the blocking buffer (20% normal goat serum, NGS, Vector Laboratories) for 1 h at room temperature and then incubated in at 4°C for long-term storage. For the antibody incubation, sections were then kept with the primary antibody at 4°C overnight. After washed with PBS at least three times, sections were incubated with the secondary antibody: dilution of Alexa 633-conjugated goat anti-rabbit antibody (1:500, Invitrogen) or Alexa 488-conjugated anti-rabbit antibody (1:250, Invitrogen) for 1 h at room temperature. After extensively washed by PBS and labeled the cell nucleus by DAPI, the free-floating sections with the positive staining by IF was captured and analyzed by a laser scanning confocal microscope (C2+, Nikon, Japan). The antibodies of IF staining are listed in **Supplementary Table 4**.

### Statistical Analysis

All statistical analyses were performed using the SPSS 20.0 software. Data were presented as means ± SEM. One-way ANOVAs followed by Tukey’s multiple comparison tests were used for the statistical analyses according to the experimental design. *P*-value < ?0.05 was considered as the significant difference for all the statistical tests. In fMRI analysis, imaging analyses were performed with ALFF images across the different bands. Statistical significance was based on an uncorrected voxel-level height threshold of *P* < 0.005, and 20 voxels were the cluster-extent threshold.

## Results

### Effects of MXYS on Depressive-Like Behaviors

We conducted a series of behavioral tests, including the SPT, TST, and FST, to examine the effects of MXYS on depressive-like behaviors. Multiple lines of evidence from our study suggest that MXYS attenuates depression-like behavior. Firstly, compared to control group, mice with CUMS treatment had shown a significant decrease in body weight (*F* = 54.99, *P* < 0.001; **Figure 1B**). While MXYS or FH administration significantly improved the body weight against CUMS treatment (**Figure 1B**). Secondly, as shown in **Figure 1C**, mice in CUMS group (*F* = 39.15, *P* < 0.001) consumed significantly less sucrose than control group according to the SPT after the CUMS procedure. However, mice with MXYS administration (*F* = 39.15, *P* < 0.001) had shown an increase in sucrose preference compared to that of mice exposed to CUMS alone, similar to the sucrose preference of FH group (*F* = 39.15, *P* < 0.001). Thirdly, one-way ANOVA shown mice with CUMS exposure significantly increased the TST immobility time compared with the control mice (*F* = 33.48, *P* < 0.001; **Figure 1D**), while a decrease was shown in the MXYS (*F* = 33.48, *P* < 0.001; **Figure 1D**) and FH (*F* = 33.48, *P* < 0.001; **Figure 1D**) groups compared with the CUMS group. Fourthly, the result of the FST was shown in **Figure 1E**. The FST immobility time of the mice in the CUMS group (*F* = 104.3, *P* < 0.001) was longer than that of the mice in the WT control group. And MXYS- (*F* = 104.3, *P* < 0.001) or FH-administration (*F* = 104.3, *P* < 0.001) mice shown a less percentage of immobility time than the CUMS-exposed mice. Notably, there was no significant difference between MXYS and FH groups in body weight and the immobility times of TST and FST.

### Effect of MXYS on BOLD-fMRI Signal in Brain Regions of Mice

**FIGURE 2:**
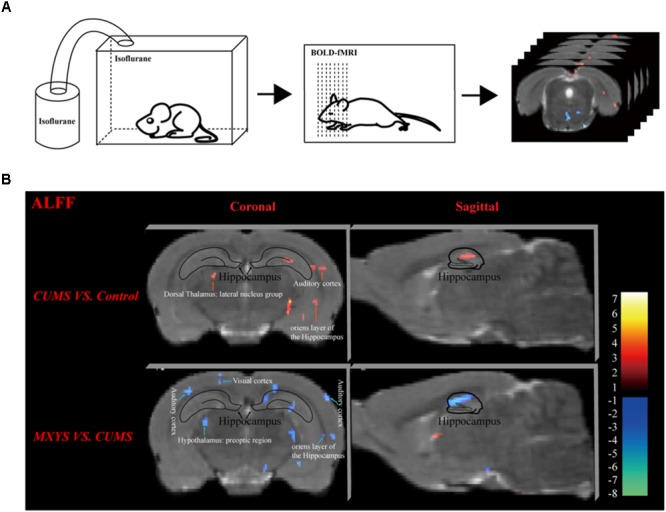
Effect of MXYS on BOLD–fMRI signal in brain regions of mice. **(A)** Schematic representation of the process for fMRI experiment. **(B)** Images of coronal and sagittal planes show the changes of BOLD–fMRI signal in the hippocampal region between different groups according to the results of the ALFF analysis. The voxel-level height threshold was *P* < 0.005 (uncorrected) and the cluster-extent threshold was 20 voxels.

As shown in **Figure 2A**, according to the standard process and the results of the ALFF analysis, a series of changes of BOLD-fMRI signal were induced by the CUMS exposure. Accompanied by depression-like behavioral changes, CUMS primarily up-regulated the BOLD signals in the limbic system (included in the auditory cortex, retrospective cortex, sensory cortex, olfactory cortex, and hippocampus) (**Table 1** and **Supplementary Figures 5, 6**), particularly in the hippocampus (**Figure 2B**). In contrast, CUMS decreased the BOLD activation in the orbital cortex, accumbens nucleus, caudate putamen, internal capsule, and amygdala (**Table 1** and **Supplementary Figures 5, 6**). Surprisingly, MXYS mice show different changes in the ALFF analysis of BOLD-fMRI. In the MXYS versus CUMS analysis, reduced activity in the brain region mainly included the hippocampus, motor cortex, prefrontal cortex, retrosplenial cortex, auditory cortex, visual cortex, sensory cortex, internal capsule, midbrain, and so on, which was the opposite of the CUMS versus control analysis. On the other hand, the signal of the amygdala, thalamus, dorsal thalamus, midbrain, and pontine: basilar part were elevated (**Table 1**, and **Supplementary Figures 7, 8**).

**Table 1:** Results of ALFF in the BOLD–fMRI Analysis.

Group	Increased structural name	*T*-average	Coordinates of the peak voxel			Decreased structural name	*T*-average	Coordinates of the peak voxel		
			*X*	*Y*	*Z*			*X*	*Y*	*Z*
CUMS vs. control	Hippocampus-CA123	7.8263	5.5422	5.9777	–5.3979	Accumbens nucleus	10.3439	–2.5853	7.0016	2.5221
	Hippocampus	7.8263	5.5422	5.9777	–5.3979	Caudate putamen	8.306	–3.755	5.9769	0.1221
	Subiculum	6.601	3.4171	3.8949	–6.3579	Orbital cortex	5.9662	–2.3312	4.0114	3.4821
	Corpus callosum	5.3351	5.2551	3.206	–3.9579	Internal capsule	5.7831	–2.148	5.7988	–0.1179
	Internal capsule	4.4931	2.9469	6.3449	–1.3179	Amygdala	5.4397	5.2452	8.983	–3.2379
	Medial prefrontal cortex	4.5567	–0.00975	0.51953	–0.1179					
	Auditory cortex	5.5281	6.1972	3.4487	–4.4379					
	Dorsal thalamus: lateral nucleus group	12.0378	–2.6265	3.5016	–4.1979					
	Midbrain: inferior colliculus	7.2675	2.244	4.6012	–8.5179					
	Nucleus around the septal area	6.2644	1.2029	5.1797	–0.8379					
	Olfactory cortex	4.5799	4.3724	6.5592	–7.7979					
	Retrosplenial cortex	6.9795	0.02985	0.77513	–2.9979					
	Sensory cortex	8.4808	5.1924	1.9251	0.6021					
	Striatum	5.3106	2.9403	6.4484	–0.8379					
MXYS vs. CUMS	Thalamus	7.4196	–1.625	7.9244	–8.9979	Fimbria of hippocampus	3.6295	4.8542	5.5192	–3.9579
	Dorsal thalamus: anterior nucleus group	3.2408	1.4834	4.5503	–1.7979	Hippocampus	7.8066	3.4072	2.9549	–5.6379
	Dorsal thalamus: lateral nucleus group	4.0186	–0.64166	6.4102	–2.7579	Hypothalamus: preoptic region	5.3071	–1.6036	7.792	–0.8379
	Midbrain: superior colliculus	4.5128	2.2077	5.243	–5.8779	Internal capsule	5.3132	–3.5785	7.1377	–2.9979
	Pontine: basilar part	7.0559	0.90761	10.0729	–8.5179	Midbrain: periaqueductal gray matter	3.4312	0.61392	5.0682	–6.5979
	Amygdala	5.4397	5.2452	8.983	–3.2379	Midbrain: tegmentum of midbrain	3.3478	1.3993	7.1984	–5.3979
						Motor cortex	4.5477	–1.1795	0.51698	–2.5179
						Lateral prefrontal cortex	5.5952	3.0244	5.8445	2.7621
						Medial prefrontal cortex	4.5567	–0.00975	0.51953	–0.1179
						Ventral prefrontal cortex	4.7738	2.8842	6.0862	3.2421
						Nucleus around the septal area	3.8544	–2.181	6.316	2.2821
						Retrosplenial cortex	6.3713	–0.24073	0.3003	–2.7579
						Auditory cortex	5.5281	6.1972	3.4487	–4.4379
						Visual cortex	8.0204	–2.2256	0.60428	–4.1979
						Sensory cortex	7.1482	6.0108	4.9985	–0.5979
						Striatum	6.6111	3.1746	5.6667	1.5621

*The voxel-level height threshold was *P* < 0.005 (uncorrected) and the cluster-extent threshold was 20 voxels.*

Obviously, we found that the hippocampus was a key brain region for BOLD signal changes. In short, compared with the control group, the hippocampal signal was elevated in the coronal (**Figure 2B**, upper left) and sagittal planes (**Figure 2B**, upper right) of CUMS group. However, it was reduced in the MXYS mice (**Figure 2B**, down) compared to the CUMS mice.

### MXYS Enhanced the BDNF Expression of the Hippocampus

As an important neurotrophin, BDNF is widely distributed within key regions in the neural circuitry, which involved in affective processing and in depression, especially in the hippocampus (Reinhart et al., 2015; Bjorkholm and Monteggia, 2016). Moreover, during the depressive disorders development, BDNF is a critical factor associated with hippocampal neuronal plasticity and neuronal survival. Therefore, we selected the BDNF as a target factor to study the mechanism through which MXYS regulates depression.

**FIGURE 3:**
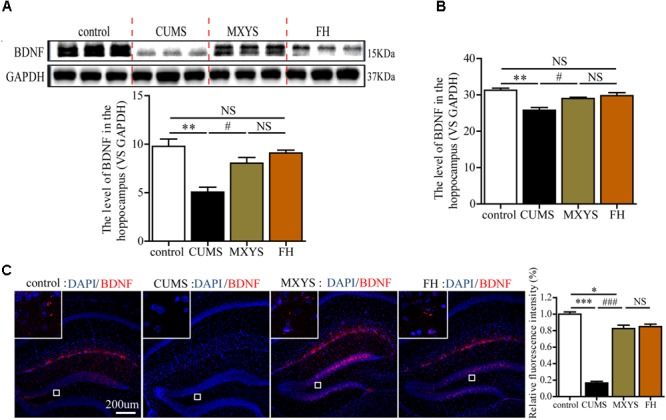
MXYS enhanced the BDNF expression of the hippocampus. BDNF expression in the hippocampus tissue of different groups was detected by western blot **(A)** and qPCR analysis **(B)**. **(C)** Representative micrographs of IF staining for the BDNF proteins (scale bar = 200?μm, 400×?magnification) in the hippocampal DG region. The data are presented as means ± SEM. ^∗^*P* < 0.05, ^∗∗^*P* < 0.01, ^∗∗∗^*P* < 0.001 versus control group; ^#^*P* < 0.05, ^###^*P* < 0.001 versus CUMS group; NS, *P* > 0.05.

In our study, western blot analysis indicated that CUMS induced down-regulation of BDNF in hippocampus compared to control mice (**Figure 3A**, *F* = 13.94, *P* < 0.01). However, the administration of MXYS or FH improved hippocampal BDNF expression to almost normal level under the effect of CUMS treatment. Similarly, qPCR analysis shows a significant increase in BDNF mRNA levels in the hippocampus of the MXYS (**Figure 3B**, *F* = 13.11, *P* < 0.05) and FH (*P* < 0.01) groups compared with the levels in the CUMS group. In addition, we next examined the expression of BDNF in the hippocampus by IF analysis. Consisted with the results of western blot and qPCR analysis, macrographs of IF staining indicated that CUMS-exposed mice had a reduction of BDNF protein in hippocampus compared to control mice (**Figure 3C**, *F* = 149.6, *P* < 0.001). And the MXYS (*P* < 0.001) and FH (*P* < 0.001) treatment mice had a significant increase in BDNF in the hippocampus against CUMS exposed. On the other hand, there was no significant difference between MXYS and FH group in the western blot, qPCR, and IF analysis (*P* > 0.05).

### MXYS Supported the Nerve Survival in the Dentate Gyrus Against the CUMS Treatment

**FIGURE 4:**
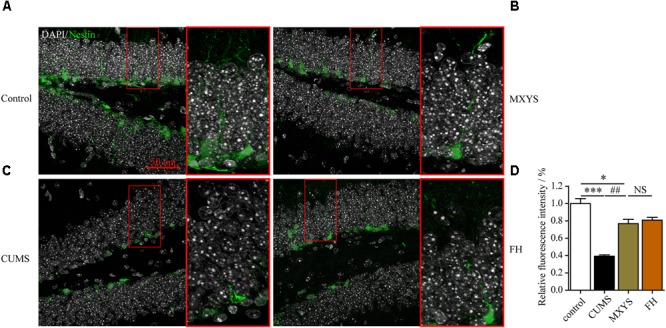
MXYS supported the Nestin-positive neurons survival in the hippocampus against the CUMS treatment. Representative micrographs of IF staining for the Nestin-positive neurons (green, scale bar = 50 μm, 400× magnification) in the hippocampal DG region of 4 groups (**A**, Control group; **B**, MXYS treatment group; **C**, CUMS group; **D**, FH treatment group). Compared with the control group, the CUMS-exposed mice displayed lower Nestin expression in the hippocampus, which were improved by the MXYS and FH administration. Data are presented as means ± SEM. ^∗^*P* < 0.05, ^∗∗∗^*P* < 0.001 versus control group; ^##^*P* < 0.01 versus CUMS group; NS, *P* > 0.05.

**FIGURE 5:**
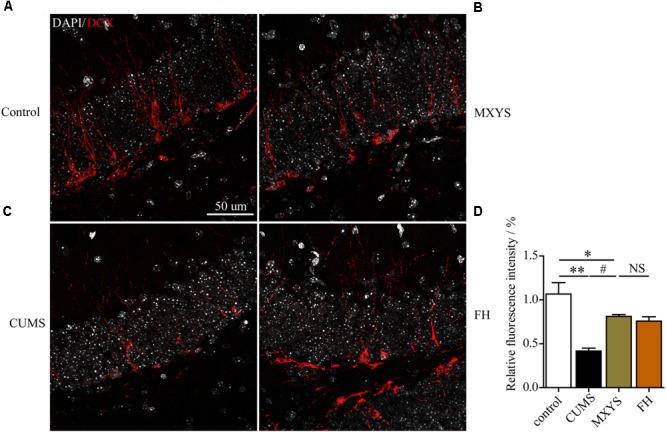
MXYS improved the hippocampal DCX-positive neurons survival against the CUMS treatment. Representative micrographs of IF staining for the DCX-positive cells (red, scale bar = 50 μm, 400× magnification) in the hippocampal DG region of 4 groups (**A**, Control group; **B**, MXYS treatment group; **C**, CUMS group; **D**, FH treatment group). Compared with the control group, the CUMS-exposed mice displayed down-regulation of DCX expression in the hippocampus, which were improved by the MXYS and FH administration. Data are presented as means ± SEM. ^∗^*P* < 0.05, ^∗∗^*P* < 0.01 versus control group; ^#^*P* < 0.05 versus CUMS group; NS, *P* > 0.05.

To assess the effect of the MXYS treatment on the generation of newly generated cells in the hippocampus of CUMS-exposed animals, we further detected immature neurons with specific markers (Nestin and DCX) in the dentate gyrus of hippocampus. As shown in **Figure 4**, the number of Nestin-positive neurons was decreased in the dentate gyrus of CUMS mice compared to the control group (*F* = 36.92, *P* < 0.001). However, it is gratifying that MXYS administration had significantly promoted neural restoration by elevating the number of Nestin-positive cells (*P* < 0.01) in the dentate gyrus. Similarly, the CUMS animals had a decrease in the numbers of DCX-positive neurons in the dentate gyrus (**Figure 5**; *F* = 13.51, *P* < 0.01). As expected, MXYS was effective in increasing the numbers of DCX-positive cells (*P* < 0.05). On the other hand, we found there was no significant difference of Nestin- or DCX-positive staining between the MXYS and FH groups.

## Discussion

The purpose of this study was to investigate the potential mechanism underlying the anti-depressive effects of MXYS on depression mice induced by CUMS. MXYS significantly ameliorated the depressive-like behaviors of mice with CUMS treatment. More importantly, mice exposed to CUMS induced hippocampal neural damage and triggered the disorders of BOLD–fMRI signal in the hippocampus, which were reversed by treatments with MXYS. Our study suggested that the MXYS administration ameliorated depression by improving the hippocampal neurogenesis and rectifying the BOLD–fMRI signals in mice with chronic stress exposure.

Chronic stress is known to be one of the risk factors for psychiatric disorders, and the animal models of mood disorders induced by stress have been widely investigated (Wolf et al., 2018). Since it effectively induces the pathophysiology of mood disorders, chronic restraint or immobilization stress is considered an appropriate paradigm for imitating psychiatric-related illnesses in rodents (Christiansen et al., 2011). Currently, CUMS is used as a classical model for the research of brain structure and neural molecules, with the reason that depressive disorders are often precipitated or exacerbated by stressful life events (Papp et al., 1991; Chourbaji et al., 2005). Based on our studies, repeated stress induced HPA hyperactivation and reduced BDNF levels in the hippocampus (Zhu et al., 2014; Jing et al., 2015; Jiang et al., 2016). According to the “neurotrophin hypothesis” (Groves, 2007), BDNF is critical for the limbic regions function associated with emotion processing and cognition in the development of depression (Nishijo et al., 2018). Furthermore, through epigenetic evidences, BDNF reduction is also regarded as the susceptibility factor for depression, which involved in the development and plasticity of hippocampal nervous (Karpova, 2014). Consistent with our previous studies, this study provides direct evidence that CUMS induced the hippocampus BDNF and neurogenesis reduction and affected behaviors associated with depression *in vivo*. The down-regulation of BDNF level in hippocampal tissue was associated with the reduction of neuronal survival, and related with several behavioral impairments, such as reduction of sucrose preference, abnormal locomotion, and activity in the water or danger environments. More damage might be associated with the reduction of BDNF in brain regions, particularly in the hippocampus, and decreased BDNF availability could also harm to neuronal growth in specific brain structure, which is critically involved in depression. In summary, depression might be promoted by the compositionality dysfunctions of neurotrophin and neuronal differentiation and proliferation in the hippocampus.

Recently, extensive communication among the brain regions with mood-regulating circuit has been observed in the onset and development of neuropsychiatric disorders, in which BOLD signal detected by fMRI plays an important indicator (Murta et al., 2015). fMRI has been widely used in investigation of the neural activity in human brain (Vanni et al., 2015; Croce et al., 2016; Kok et al., 2018), but few fMRI studies focus on the animal models of psychiatric diseases. Over the past decade, the rodent brain template was constructed by scientists in order to guide for human brain studies and standardized the animal fMRI analysis procedure (Papp et al., 1991). The digital mice brain atlas was constructed from the fifth edition of the rat brain in stereotaxic coordinates by Paxinos and Watson, and published in Human Brain Mapping recently (Nie et al., 2013). Based on our previous fMRI studies, CUMS-induced depressive behaviors accompanied with up-regulation of BOLD signals in mouse brain regions, primarily in the limbic system, such as the cortex, hippocampus, and corpus calcium, depending on the ALFF analysis (Huang et al., 2017). Recently, we further demonstrated that hippocampus was connected with the prefrontal cortex and basolateral amygdala to maintain the neural circuitry in CUMS-induced depression mice model, which were associated with BOLD–fMRI signals (Huang et al., 2018). Consistent with the previous cognitions, the hippocampus has critical roles in the development and maintenance of depression (Lee et al., 2013). Thus, depression may be driven by dysregulated function across multiple brain regions based on emerging visual evidence from humans and animals. Unfortunately, up to now, few studies are using the fMRI technology to evaluate the neuroprotection effects of herbal medicine.

As the classic traditional Chinese herbal medicines, Xiaoyaosan and its modified formulas have been widely used to cure the patients with mental disorders in China for centuries. They are generally recognized by clinical researchers as a safe and effective prescription for the therapy of depressive disorders (Jing et al., 2015; Li et al., 2017). Similarly to the Xiaoyaosan, our findings also have shown the reliable antidepressant effects of MXYS in mice. As shown in the present study, MXYS treatment not only significantly ameliorated the body weight and depressive-like behaviors in mice after exposure to CUMS, but also up-regulated the BDNF levels and restored neurogenesis in hippocampus. Moreover, according to the fMRI detection, MXYS could rectify the CUMS-induced excessive BOLD activation of hippocampus, auditory cortex, retrospective cortex, and sensory cortex. Notably, BOLD–fMRI signal in amygdala, which was suppressed by CUMS administration, was also partially rectified by MXYS. Interestingly, most of these brain regions are involved in the mood-regulating circuits, especially the hippocampus and amygdala, suggesting dysfunction of these circuits in the CUMS-induced depressive mice, and MXYS can adjust the key regions of the mood-regulating circuits. It can be used to guide the clinical treatment of depression and MDD patients.

It is important to note that MXYS consists of several Chinese herbs, and its composition is complex. Our team has determined the composition of MXYS using high-performance liquid chromatography (HPLC) and identified seven of the main compounds, including paeoniflorin, ferulic acid, paeonol, schizandrin, emodin, butenolide I, and curcumin in MXYS samples. Based on these results, the complicated compounds contained in MXYS may be responsible for its anti-depressive effects. However, until now, the mechanisms underlying the formulas remain unclear due to the complex composition and multiple targets of these medicines. In the next step, we will devote our attention to clarifying the potential active components with the antidepressant properties in this prescription. On the other hand, although the preliminary effects of the hippocampal neurogenesis of MXYS administration have been described in the present study, further investigation will be needed to confirm the underlying mechanisms.

**FIGURE 6:**
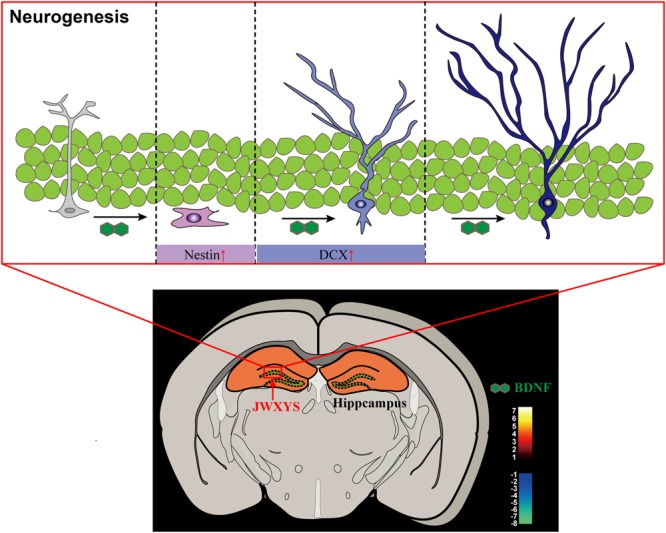
Graphical abstract of the mechanism of MXYS exerts anti-depressive effects through ameliorating BOLD-signal and hippocampal neurogenesis in mice.

The present study provides evidence for the anti-depressive mechanism of MXYS in mice exposed to CUMS, as shown in **Figure 6**. As far as we know, this is the first study to demonstrate the brain functional effects of MXYS depending on BOLD-based fMRI analysis in CUMS mice model. These findings, together with our previous studies, reinforce the notion that emotional behaviors in rodents are BOLD activity-dependent, and that MXYS is a potential therapeutic method for attaining positive emotional states through rectifying brain BOLD signals function and hippocampal neurogenesis. These findings provide a new perspective for the general concept of TCM in the regulation of brain functions associated to depression.

## Author Contributions

ZL, LG, and GW conceived and designed the study. PH constructed the animal model. PH, TG, and ZD performed the fMRI experiments. CZ and YL performed the RT-PCR experiment. GD and PH performed the IF staining. LG, ZD, BL, and PH analyzed the data and wrote the manuscript. ZL, LG, SH, and BL contributed reagents, materials, and analytical tools.

## Conflict of Interest Statement

The authors declare that the research was conducted in the absence of any commercial or financial relationships that could be construed as a potential conflict of interest. The handling Editor declared a shared affiliation, though no other collaboration, with one of the authors ZD at time of review.
