# Corrigendum: Modified Xiaoyaosan (MXYS) Exerts Anti-depressive Effects by Rectifying the Brain Blood Oxygen Level-Dependent FMRI Signals and Improving Hippocampal Neurogenesis in Mice

**DOI:** 10.3389/fphar.2018.01420

**Published:** 2018-12-03

**Authors:** Lei Gao, Peng Huang, Zhaoyang Dong, Tingting Gao, Shaohui Huang, Chuying Zhou, Yuling Lai, Guanghui Deng, Bin Liu, Ge Wen, Zhiping Lv

**Affiliations:** ^1^School of Traditional Chinese Medicine, Southern Medical University, Guangzhou, China; ^2^School of Nursing, Guangzhou University of Chinese Medicine, Guangzhou, China; ^3^Zhujiang Hospital, Southern Medical University, Guangzhou, China; ^4^Nanfang Hospital, Southern Medical University, Guangzhou, China

**Keywords:** depression, FMRI, traditional Chinese medicine, hippocampus, blood oxygen level-dependent

There is an error in the Funding statement. The correct number for the National Natural Science Foundation of China is 81230085. The authors apologize for this error and state that this does not change the scientific conclusions of the article in any way. The original article has been updated.

## Conflict of Interest Statement

The authors declare that the research was conducted in the absence of any commercial or financial relationships that could be construed as a potential conflict of interest.

