# Supplementary material for: Modified Xiaoyaosan (MXYS) Exerts Anti-depressive Effects by Rectifying the Brain Blood Oxygen Level-Dependent fMRI Signals and Improving Hippocampal Neurogenesis in Mice
Source: Front Pharmacol. 2018 Sep 28;9:1098. doi: 10.3389/fphar.2018.01098 (PMC6173122; doi:10.3389/fphar.2018.01098)
Supplement: Supplementary file 1 [file Data_Sheet_1.PDF]

**Modified Xiaoyaosan (MXYS) exerts anti-depressive effects by rectifying the brain Blood oxygen level-dependent fMRI signals and improving hippocampal neurogenesis in mice**

## Supplementary Material

## Preparation of Modified Xiaoyaosan (MXYS)

MXYS is an empirical prescription used by Professor Lv Zhiping for the treatment of depression. The composition of MXYS is as follows: Radix Bupleuri (15g), Radix Angelicae Sinensis (15g), Radices Paeoniae Alba (15g), Rhizoma Atractylodis Macrocephalae (15g), Rhizoma Acori Tatarinowii (12g), Curcuma Aromatica (15g), Caulis polygoni multiflori (30g), Fructus Schisandrae Chinensis (10g), Semen Ziziphi Spinosae (20g) and Os Draconis (30g) (listed in **Supplementary Table 1**)( Bi et al.). All of the raw herbs were purchased from Nan fang Hospital and processed into a no decocted extract in EFONG Pharmaceutical company (Guangdong, China) in accordance with the Regulation on Processing of Traditional Chinese Medical Herbal Pieces, production batch number: J1606007. The process used to produce the no decocted MXYS was described in **Supplementary Fig. 1.**

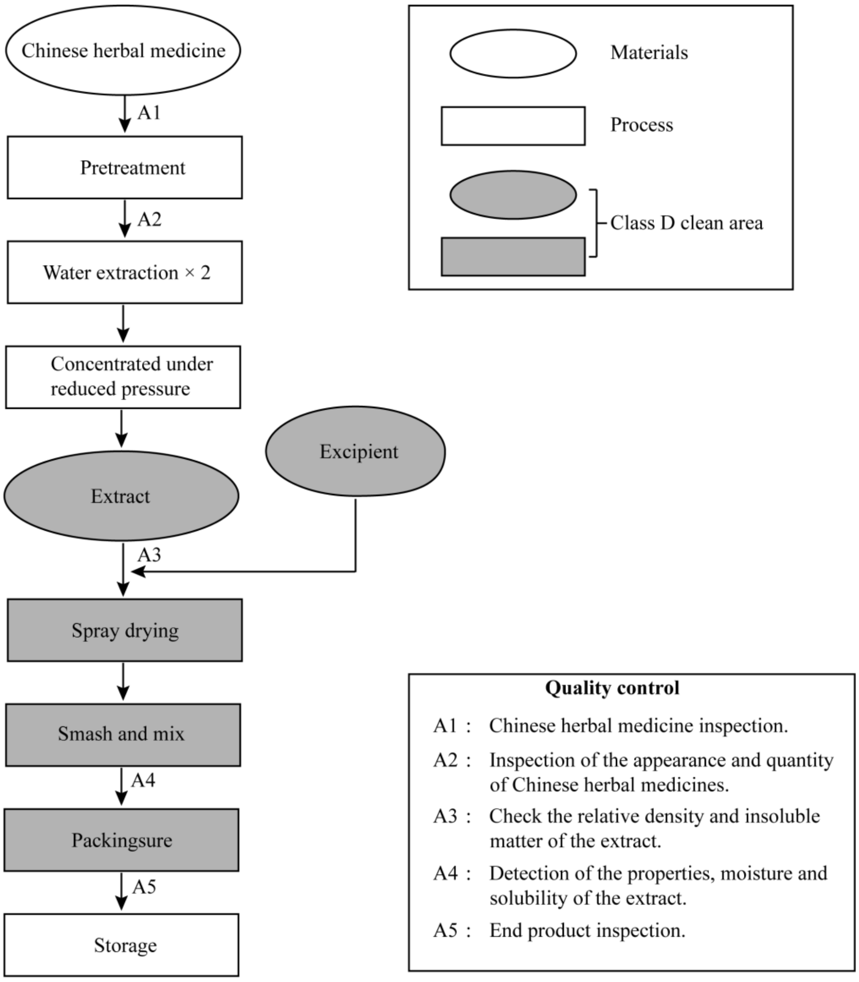


**Supplementary Figure 1. The process used to produce the no decocted MXYS**

According to the formula, the same amount of the extract is converted into a granule. The quality of MXYS Granule is controlled by the regular items under the 2010 edition of the Pharmacopoeia granules. The drug is packaged in a medicinal composite film, which is in Line with the pharmaceutical packaging specifications. In our experiments, the drug dose was converted according to the human and animal equivalent dose formula.

**Quality control of modified Xiaoyaosan (MXYS) by high performance liquid chromatography (HPLC)**

1. Chemicals and materials

HPLC-grade acetonitrile was purchased from Merck co., Ltd (Darmstadt, Germany). Methanol, ethanol and phosphoric acid were of analytical grade and purchased from Guangzhou chemical reagents factory (Guangzhou, China). Deionized water obtained by Millipore Milli-Q water system (Bedford, USA).

Standards of paeoniflorin (batch No. 110736-201438), ferulic acid (batch No. 110773-201318), paeonol (batch No.110708-201407), schizandrin (batch No.110857-201211), emodin (batch No.110756-20151), butenolide I(batch No.111975-201501) and curcumin (batch No.110823-201405) were purchased from the National Institutes for Food and Drug Control (Beijing, China).

Medications listed in table S1 were purchased from Nan fang Hospital. Ten MXYS samples were prepared by our laboratory, and the batch numbers were 20170715-01、20170715-02、20170715-03、20170716-01、20170716-02、20170716-03、20170717-01、20170717-02、20170717-03.

**Supplementary Table 1. Composition of Modified Xiaoyaosan (MXYS)**

| Medicinal plant | Amount (g) |
|---|---|
| *Radix Bupleuri* | 15.0 |
| *Radix Angelicae Sinensis* | 15.0 |
| *Radix Paeoniae Alba* | 15.0 |
| *Rhizoma Atractylodis Macrocephalae* | 15.0 |
| *Rhizoma Acori Tatarinowii* | 12.0 |
| *Curcuma Aromatica* | 15.0 |
| *Caulis polygoni multiflori* | 30.0 |
| *Fructus Schisandrae Chinensis* | 10.0 |
| *Semen Ziziphi Spinosae* | 20.0 |
| *Os Draconis* | 30.0 |

2. Instrumentation and chromatographic conditions

Chromatographic analysis was performed on an Agilent 1100 infinity HPLC-DAD system (Palo Alto, USA) equipped with a quaternary pump (G1311A) and a diode array detector (G1215B). The chromatographic data were processed with Agilent ChemStation. Sample separation was carried on an Atlantis ®d C18 column (250 mm × 4.6 mm, 5 μm) at 25 °C. The mobile phase was composed of solvent A (water-0.2% phosphoric acid) and solvent B (acetonitrile), and the flow rate was kept at 1.0 mL/min. The injection volume was 10 μL and the detection wavelength was set at 230 nm from 0 min to 40 min and set at 298 nm from 40 min to 140 min. The gradient elution was as follows (**Supplementary Table 2**):

**Supplementary Table 2. The gradient elution program**

| Time (min) | A(%) | B(%) |
|---|---|---|
| 0 | 90 | 10 |
| 20 | 89 | 11 |
| 60 | 75 | 25 |
| 70 | 62 | 38 |
| 100 | 50 | 50 |
| 115 | 56 | 44 |
| 125 | 58 | 42 |
| 140 | 58 | 42 |

3. Preparation of standard solutions, medications solutions and modified XYS samples

Stock solutions of seven reference standards were prepared at a concentration of 0.5 mg/mL in methanol, respectively. The mixed standard working solutions of lower concentration (0.05mg/mL) was obtained by appropriate diluting stock solutions to desired concentrations. All the stock and working solutions were stored at 4 ℃and filtered through 0.45μm nylon membranes before injection.

Each kind of the herbs of MXYS was weighed 5g and extracted with methanol 25 mL at room temperatureby ultrasonic extraction for 20 min and filtered through 0.45μm nylon membranes before injection.

According to the herb proportion in the prescription, the mixture herbal formula was weighed 10g and extracted with methanol 25 mL at room temperature by ultrasonic extraction for 20 min and filtered through 0.45μm nylon membranes before injection.

4. Precision, stability and repeatability validation

The sample solution (batch No: 20170715-01) was injected for 5 times continuously. Paeoniflorin was used as the correction peak. The results showed that the RSD of the relative retention time of the common peaks was 0.82% and the relative peak area RSD was 1.27%.Stability was tested by analyzing the sample solutions (batch No: 20170715-01) at 0, 2.5, 5, 10, and 24 h. Accuracy was evaluated as percentage recovery by using paeoniflorin as the correction peak. The results showed that the RSD of the relative retention time of the common peaks was 1.28% and the relative peak area RSD was 1.47%.The repeatability was determined by analyzing nine independently prepared solutions of the MXYS sample. The results showed that the RSD of the relative retention time of the common peaks was 1.78% and the relative peak area RSD was 2.82%.The above results indicated that the developed assay was reproducible and reliable with the good accuracy for the quantification in samples.

5. Establishment of fingerprint

The standard solution, medicinal solution and sample solution were injected into HPLC. Seven characteristic peaks were identified through comparative analysis, which were determined as paeoniflorin (1), ferulic acid (2), paeonol (3), schisandrin (4), curcumin(5), emodin (6) and butenolideI(7)(Figure1 and 2).

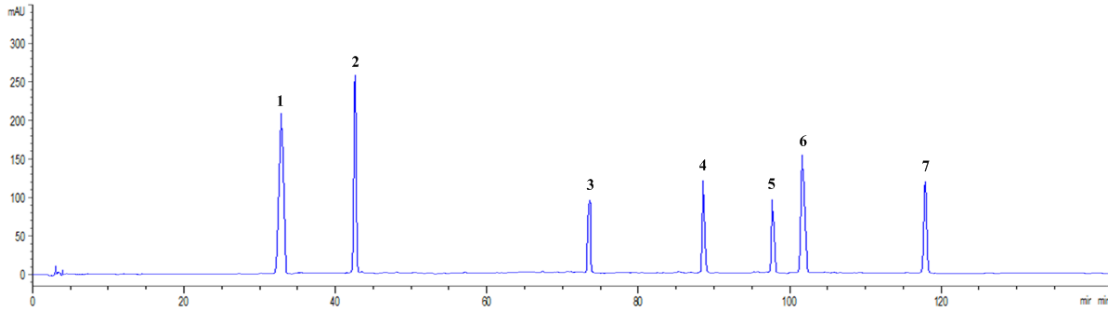


**Supplementary Figure 2.** **HPLC chromatogram of mixed standards**

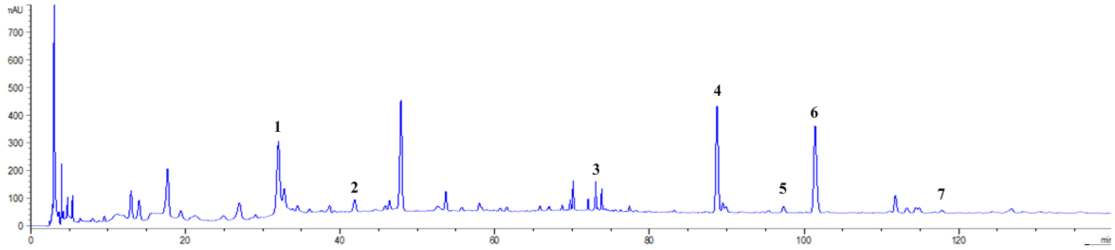


**Supplementary Figure 3. HPLC chromatogram of MXYS sample**

The Similarity Evaluation System for Chromatographic Fingerprint of TCM (2004 A edition) was used to evaluate the similarities of the 9 batches of MXYS. After peak-picking, template-matching process, the peaks in the spectra were matched

automatically (**Supplementary Figure 3 and Table 3**). The reference template was set finally for spectra peak difference and entire similarity evaluation. The similarities of repeatability was greater than 0.96. The results showed that the preparation process of MXYS was reasonable.

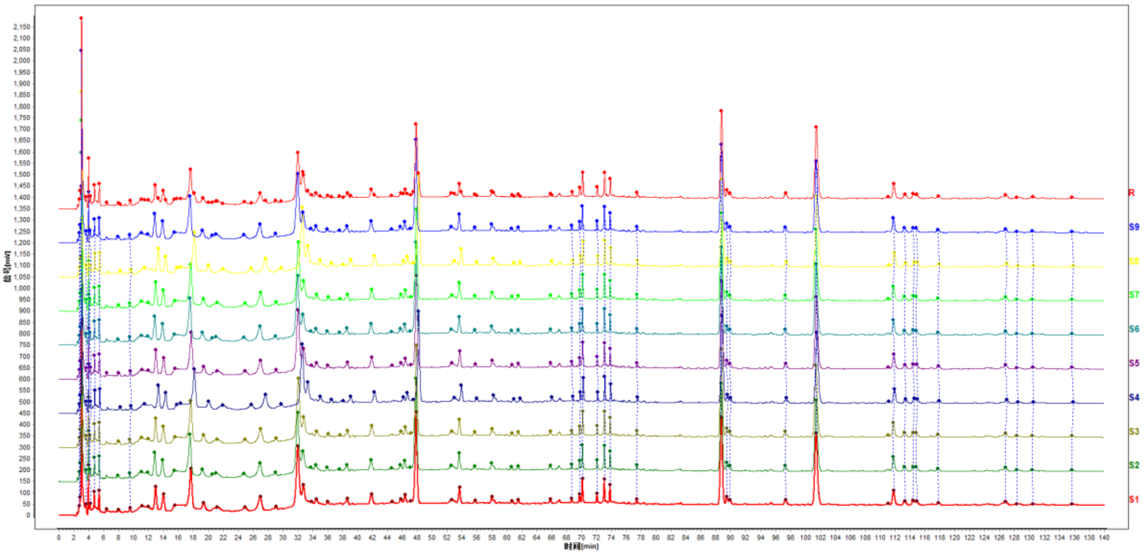


**Supplementary Figure 4. Similarity analysis of chromatographic MXYS samples**

**Supplementary Table 3. Comparability results of reproducibility of MXYS samples**

| | S1 | S2 | S3 | S4 | S5 | S6 | S7 | S8 | S9 | Reference Fingerprint |
|---|---|---|---|---|---|---|---|---|---|---|
| S1 | 1.000 | 0.997 | 0.988 | 0.998 | 0.998 | 0.966 | 0.955 | 0.996 | 0.965 | 0.996 |
| S2 | 0.997 | 1.000 | 0.986 | 0.996 | 0.996 | 0.965 | 0.954 | 0.997 | 0.963 | 0.995 |
| S3 | 0.988 | 0.986 | 1.000 | 0.988 | 0.987 | 0.972 | 0.944 | 0.985 | 0.953 | 0.989 |
| S4 | 0.998 | 0.996 | 0.988 | 1.000 | 0.998 | 0.966 | 0.956 | 0.996 | 0.962 | 0.996 |
| S5 | 0.998 | 0.996 | 0.987 | 0.998 | 1.000 | 0.966 | 0.955 | 0.997 | 0.965 | 0.996 |
| S6 | 0.966 | 0.965 | 0.972 | 0.966 | 0.966 | 1.000 | 0.925 | 0.963 | 0.948 | 0.975 |
| S7 | 0.955 | 0.954 | 0.94 | 0.95 | 0.95 | 0.92 | 1.00 | 0.95 | 0.93 | 0.964 |

| | | | | | | | | | | |
|---|---|---|---|---|---|---|---|---|---|---|
| | | | 4 | 6 | 5 | 5 | 0 | 5 | 7 | |
| S8 | 0.996 | 0.997 | 0.985 | 0.996 | 0.997 | 0.963 | 0.955 | 1.000 | 0.963 | 0.995 |
| S9 | 0.965 | 0.963 | 0.953 | 0.962 | 0.965 | 0.948 | 0.937 | 0.963 | 1.000 | 0.979 |
| Reference Fingerprint | 0.996 | 0.995 | 0.989 | 0.996 | 0.996 | 0.975 | 0.964 | 0.995 | 0.979 | 1.000 |

**Supplementary Table. 4** List of antibodies used for immunofluorescence, western blot and in situ hybridization experiments

| Antigen | Immunogen | Manufacturer | Application | Dilution |
|---|---|---|---|---|
| GAPDH | Glyceraldehyde 3-phosphate dehydrogenase (GAPDH) is an ubiquitous glycolytic enzyme present in reasonably high levels in almost all tissues. | Millipore, (Cat. #ABS16), rabbit monoclonal | WB | 1:2000 |
| BDNF | Synthetic peptide within Human BDNF aa 150 to the C-terminus. The exact sequence is proprietary. | Abcam, (Cat. #ab108319), rabbit monoclonal | WB<br>IF | 1:1000<br>1:200 |
| Nestin | Nestin is expressed in both mature and precursor neuronal and glial cells, as well as in the developing brain and in the brain and spinal cord following damage, Nestin is widely accepted as a marker of neural stem/progenitor cells. | CST, (Cat. #4760), Mouse monoclonal | IF | 1:400 |
| DCX | Doublecortin is a microtubule associated protein that stabilizes and bundles microtubules. | CST, (Cat. #4604), rabbit monoclonal | IF | 1:300 |

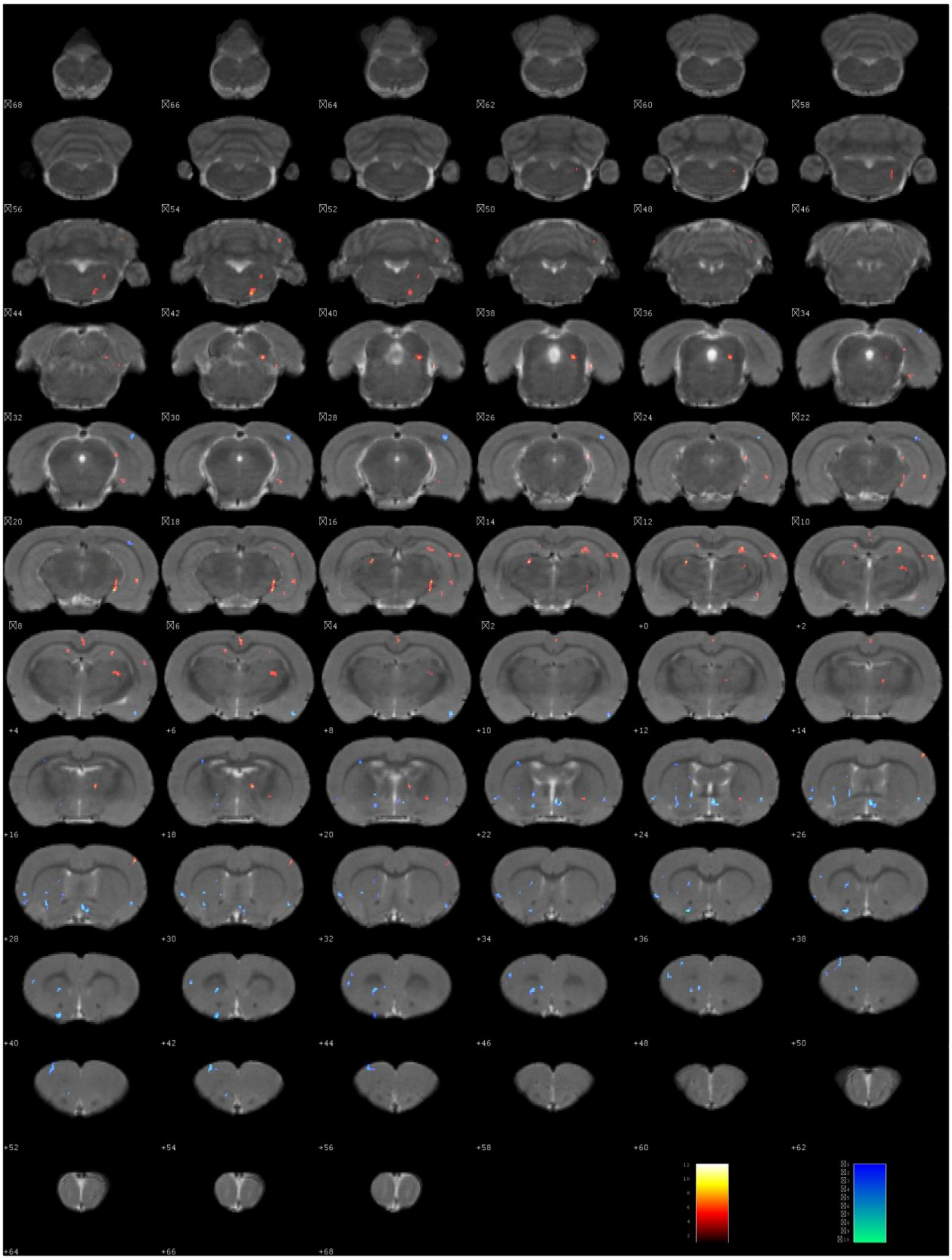

**Supplementary Figure 5.** Statistical results of BOLD-fMRI signals in coronal planes of the CUMS group compare with control group. The voxel-level height threshold was $P < 0.005$(uncorrected) and the cluster-extent threshold were 20 voxels.

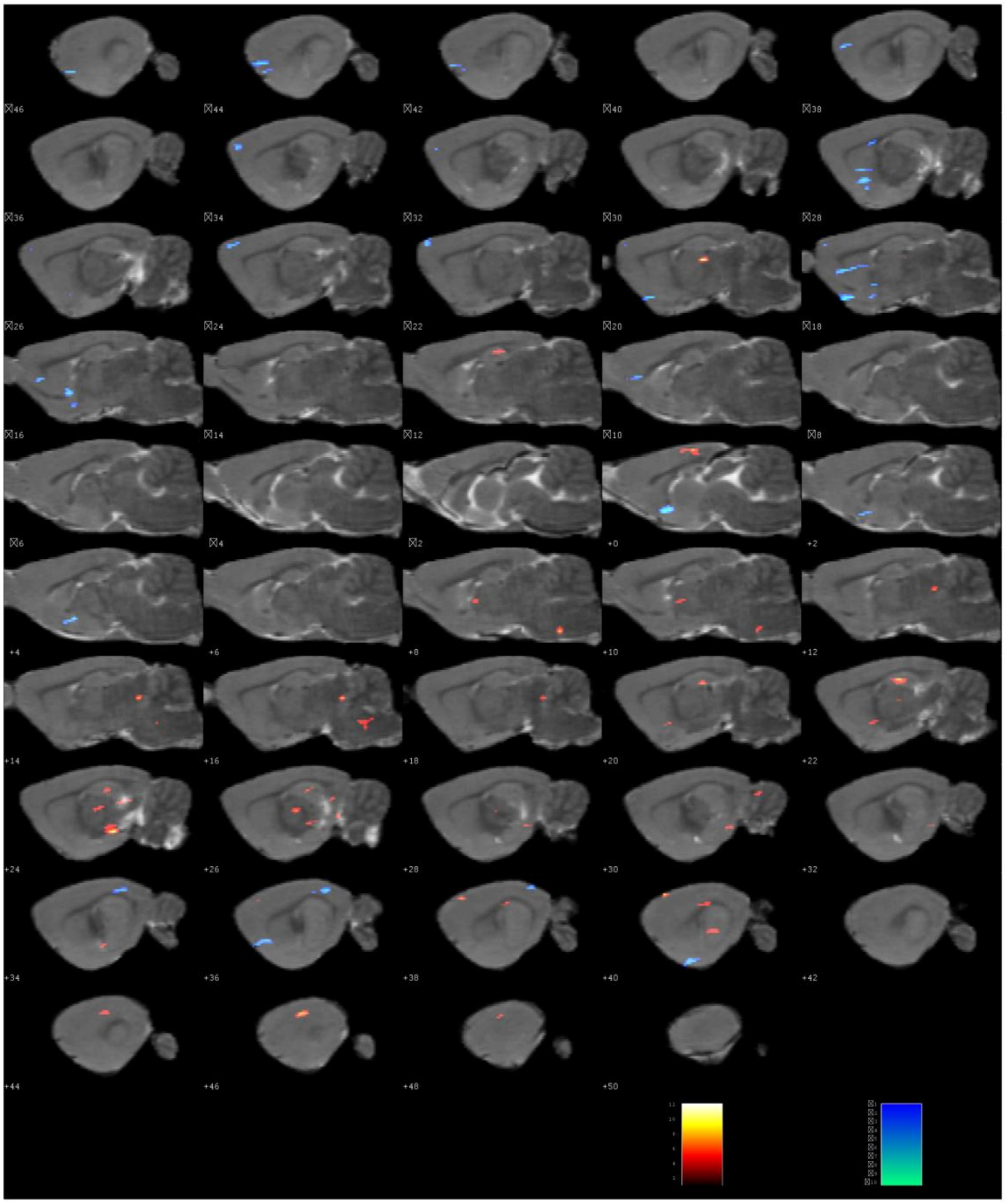

**Supplementary Figure 6.** Statistical results of BOLD-fMRI signals in sagittal planes of the CUMS group compare with control group. The voxel-level height threshold was $P < 0.005$(uncorrected) and the cluster-extent threshold were 20 voxels.

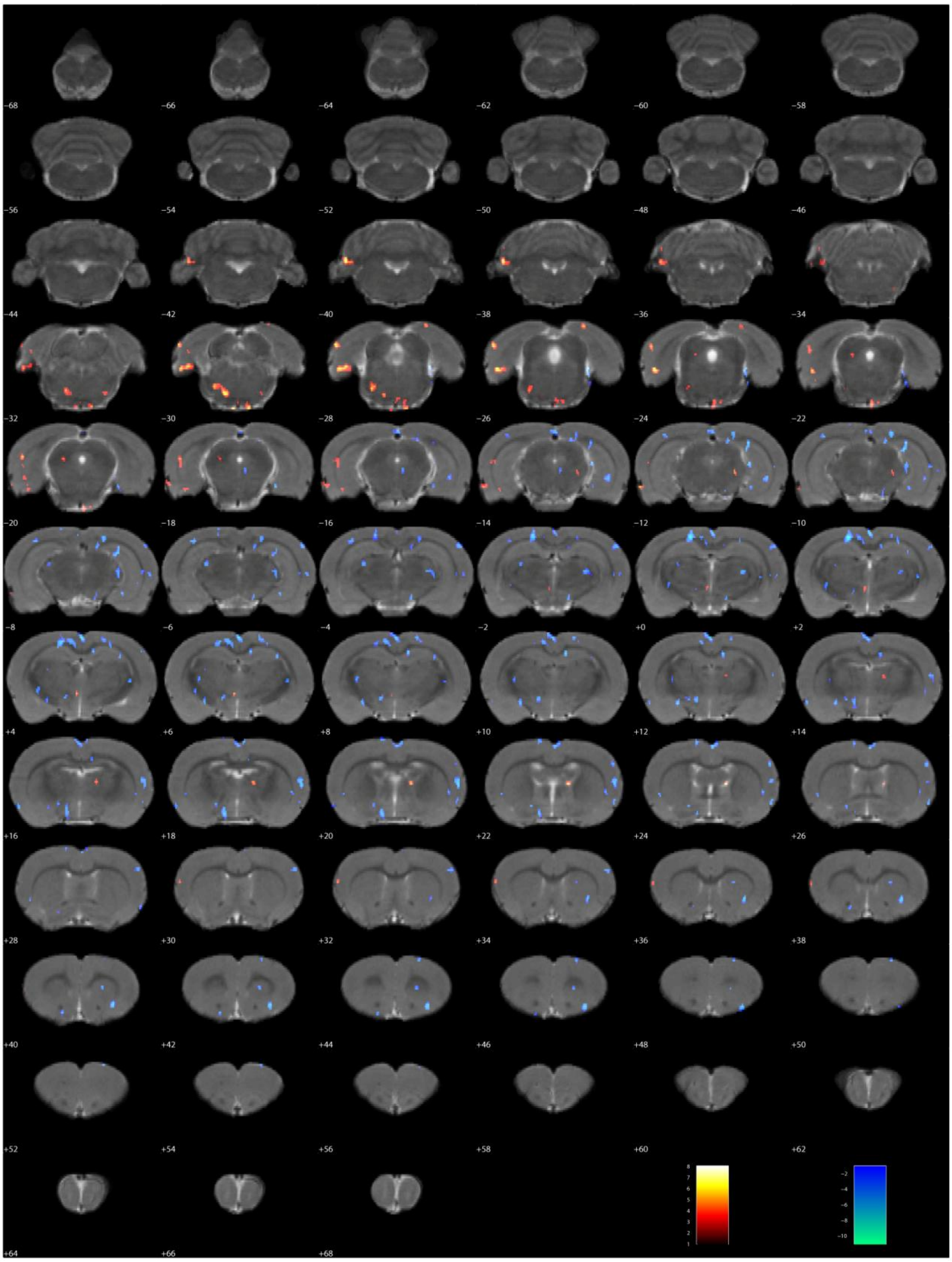

**Supplementary Figure 7.** Statistical results of BOLD-fMRI signals in coronal planes of the MXYS group compare with CUMS group. The voxel-level height threshold was $P < 0.005$(uncorrected) and the cluster-extent threshold were 20 voxels.

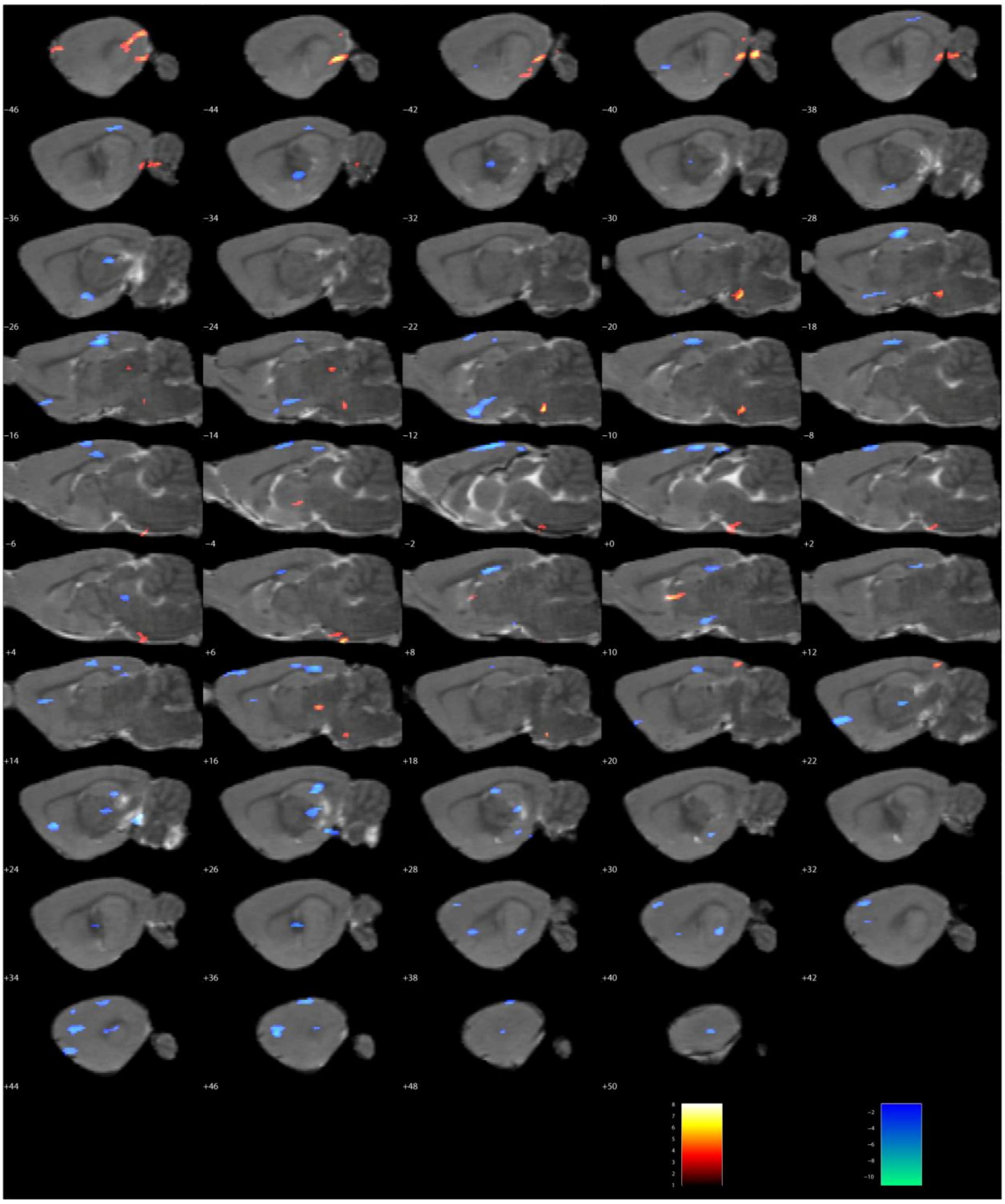

**Supplementary Figure 8.** Statistical results of BOLD-fMRI signals in sagittal planes of the MXYS group compare with CUMS group. The voxel-level height threshold was $P < 0.005$(uncorrected) and the cluster-extent threshold were 20 voxels.

# References:

[Bi Yan-meng, Wen Jin-feng, Liu Yuan, Zhao Xiao-hua, Gao Ting-ting, Qu Shan-shan, Huang Yong, and Lyu Zhi-ping. (2018). Clinical Observation of Integrated Chinese-western Therapy in Treating Depression. *Journal of Guangzhou University of Traditional Chinese Medicine*. Vol. 35, No. 5, 1-8. Chinese. ]
